# Investigating the Life Expectancy and Proteolytic Degradation of Engineered Skeletal Muscle Biological Machines

**DOI:** 10.1038/s41598-017-03723-8

**Published:** 2017-06-19

**Authors:** Caroline Cvetkovic, Meghan C. Ferrall-Fairbanks, Eunkyung Ko, Lauren Grant, Hyunjoon Kong, Manu O. Platt, Rashid Bashir

**Affiliations:** 1 Department of Bioengineering, University of Illinois at Urbana-Champaign, Urbana, Illinois 61801 USA; 2 Micro and Nanotechnology Laboratory, University of Illinois at Urbana-Champaign, Urbana, Illinois 61801 USA; 3 Wallace H. Coulter Department of Biomedical Engineering, Georgia Institute of Technology and Emory University, Atlanta, 30332 Georgia USA; 4 Department of Chemical and Biomolecular Engineering, University of Illinois at Urbana-Champaign, Urbana, Illinois 61801 USA; 5 Carle Illinois College of Medicine, University of Illinois at Urbana-Champaign, Urbana, Illinois 61801 USA

## Abstract

A combination of techniques from 3D printing, tissue engineering and biomaterials has yielded a new class of engineered biological robots that could be reliably controlled via applied signals. These machines are powered by a muscle strip composed of differentiated skeletal myofibers in a matrix of natural proteins, including fibrin, that provide physical support and cues to the cells as an engineered basement membrane. However, maintaining consistent results becomes challenging when sustaining a living system *in vitro*. Skeletal muscle must be preserved in a differentiated state and the system is subject to degradation by proteolytic enzymes that can break down its mechanical integrity. Here we examine the life expectancy, breakdown, and device failure of engineered skeletal muscle bio-bots as a result of degradation by three classes of proteases: plasmin, cathepsin L, and matrix metalloproteinases (MMP-2 and MMP-9). We also demonstrate the use of gelatin zymography to determine the effects of differentiation and inhibitor concentration on protease expression. With this knowledge, we are poised to design the next generation of complex biological machines with controllable function, specific life expectancy and greater consistency. These results could also prove useful for the study of disease-specific models, treatments of myopathies, and other tissue engineering applications.

## Introduction

The modular and scalable architecture of skeletal muscle tissue makes it an ideal power supply for producing force and locomotion – on a range of length and time scales – in engineered living systems and biological actuators. Skeletal muscle is the primary actuating source for mammals, which are evolutionarily considered to be the highest life forms and capable of many complex behaviors and high-level functionalities^1–3^. A combination of techniques from the fields of 3D printing, tissue engineering, and biomaterials has recently yielded a new class of millimeter-to-centimeter scale skeletal muscle-powered biological robots (‘bio-bots’) capable of dynamic and adaptive responses, reliable force production and untethered locomotion upon applied electrical^4^ or optical^5, 6^ signals. This forward-engineered biomaterial-muscle platform is ideal for introducing different cell types and biomaterials and permits control over physical, mechanical, biological, and biochemical cues^2^.

*In vivo*, skeletal muscle is supported by a basal lamina containing layers of connective tissue that provide a barrier from physical stress and damage^7, 8^. A combination of extracellular matrix (ECM) proteins, including the natural hydrogel of fibrin, acts as a bioinspired basement membrane mimic that provides cues and physical support to the cells in the engineered solid muscle strip of the bio-bots^4^. Though synthetic polymers have been used in the fabrication of some skeletal muscle constructs, the viscoelastic mechanical properties of natural proteins such as fibrin can be tuned to those of native connective tissue or skeletal muscle, rendering it a useful scaffold material for engineering a myriad of tissue types^9, 10^. However, maintaining consistent results becomes challenging when sustaining a living cellular system *in vitro* for weeks or months. Skeletal muscle must be preserved in a differentiated state at environmental conditions^2^. Moreover, the system is subject to degradation by cell-secreted proteases that can break down the mechanical integrity of the ECM in tissue and thus eventually lead to device failure, despite the presence of some protease inhibitors^4^.

The polymerization of fibrin into a 3D branching network is actively and equally opposed in a physiological equilibrium by fibrinolysis; despite cross-linking, the protein is extremely susceptible to degradation by cell-secreted enzymatic proteases^11, 12^ (Supplementary Fig. S1). Plasmin is a serine protease secreted by many different cells to rapidly degrade fibrin^13, 14^. In skeletal muscle, inhibition of plasmin can result in fibrin build up and reduced myoblast fusion, differentiation and regeneration^15^. Furthermore, other proteases secreted during myoblast differentiation, including cysteine cathepsins and matrix metalloproteinases (MMPs), must also be considered as contributors to fibrin network destabilization^16–18^. Cysteine cathepsins are powerful lysosomal proteases that degrade intracellular and extracellular matrix proteins and include the most powerful mammalian collagenase and elastase^19–23^; their upregulation during myogenic differentiation has been well documented^16^. MMPs are another group of ECM-degrading enzymes involved in remodeling and maintenance of the ECM in both normal and pathological states^17, 18^. Specifically, MMP-2 and MMP-9 have been seen to be involved with skeletal muscle ECM remodeling in muscle satellite cell migration and differentiation, and are differentially regulated by cytokines and growth factors during these processes. While MMP-9 has been implicated in the early stages of regeneration and pre-fusion migration, MMP-2 activation occurs at later stages of myofiber regeneration^17, 18, 24, 25^.

Here we examine the life expectancy and breakdown of engineered skeletal muscle bio-bots by loss of form and function, as a result of degradation by three major classes of proteases: plasmin, cathepsin L (CatL), and MMPs (including MMP-2 and MMP-9). We first examined the effect of adding a serine protease inhibitor, aminocaproic acid (ACA), on the maintenance of machine life expectancy and structural integrity. We also demonstrate the use of gelatin zymography, a substrate gel electrophoresis technique, to determine the effects of differentiation time, inhibitor concentration and electrical stimulation on temporal cysteine cathepsin and MMP expression within muscle strips. An experimental window under two weeks allowed for an increased understanding of the system on a shorter time scale. Finally, we demonstrate the ability to measure CatL and MMP activity while modifying certain design parameters involved in bio-bot fabrication. With an understanding of these cell-secreted proteases and concomitant matrix degradation, we are poised to design the next generation of machines with controllable gain and loss of function. Our studies also have important applications in skeletal muscle based tissue engineering, muscles-on-a-chip and related fields of studies.

## Results

### Development and Maturation of Skeletal Muscle Bio-Bots

The freestanding bio-bot consisted of an engineered skeletal muscle strip coupled to a fabricated hydrogel skeleton, or a scaffold consisting of a flexible beam connected by two rigid pillars – a design inspired by the musculoskeletal physiology of tendon and bone in which the force from a contractile muscle is transmitted to a bone through an adjoining tendon^4^. We used stereolithographic 3D printing, a rapid manufacturing technique that prints a structure with customizable geometric and mechanical parameters in a layer-by-layer fashion^26–30^, to polymerize the bio-bot skeleton from a synthetic biocompatible hydrogel.

**Figure 1:**
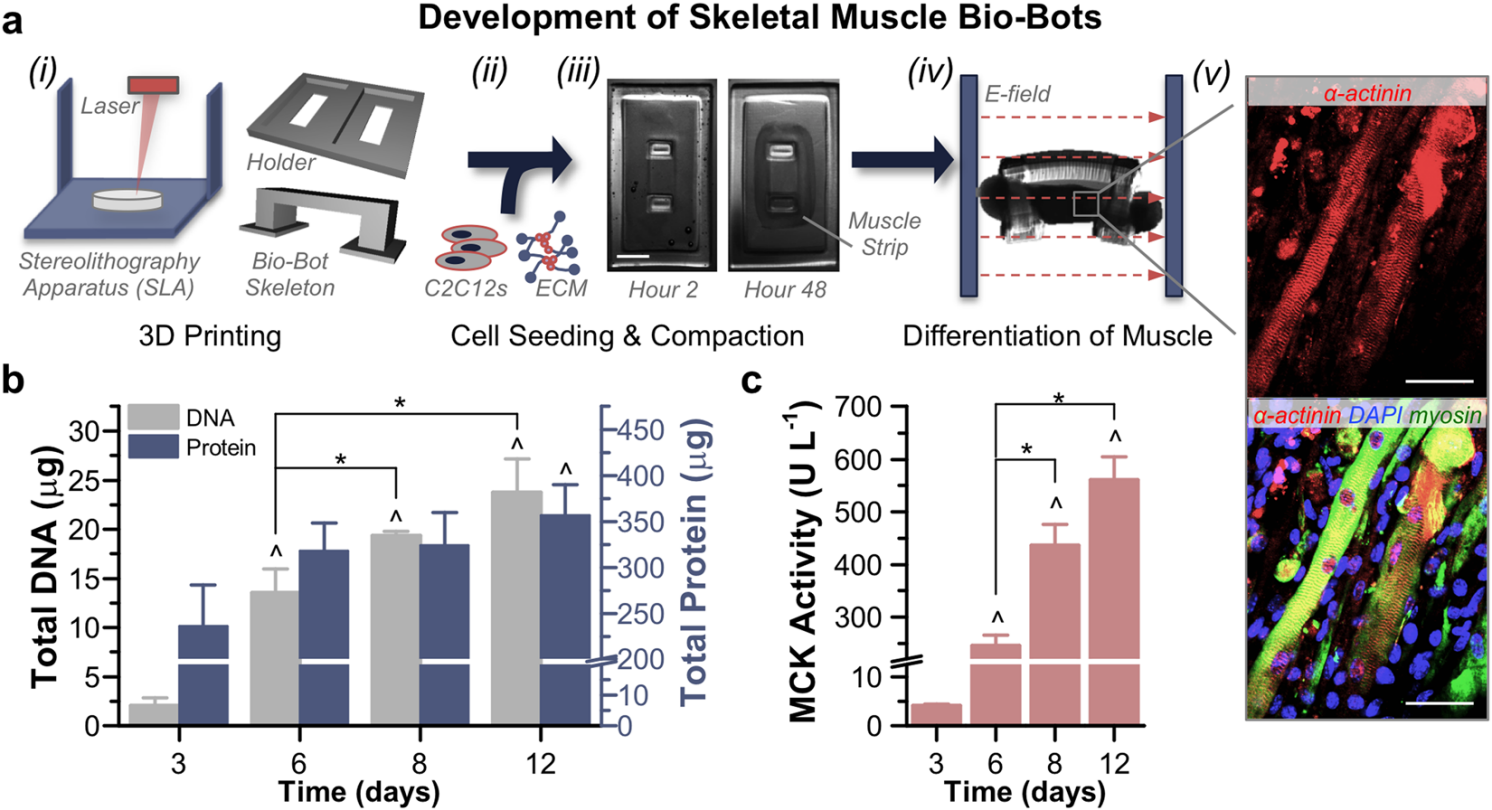
Development and Differentiation of Skeletal Muscle Bio-Bots. (**a**) Modular bio-bots were assembled and differentiated in a stepwise manner. *(i)* A Stereolithography apparatus (SLA) was used to 3D print a millimeter-scale hydrogel skeleton and holder. *(ii)* C2C12s were mixed with a liquid solution of ECM proteins that included fibrinogen, thrombin and Matrigel. *(iii)* When added to the holder, the cell-gel solution compacted to form a solid muscle strip. Scale bar, 2 mm. *(iv)* The freestanding bio-bot (consisting of the muscle strip coupled to the hydrogel skeleton) could be released from the holder and subjected to electrical or optical stimulation. *(v)* Immunostaining revealed the presence of striated and multinucleated myotubes (α-actinin, red; MF-20 myosin, green; DAPI nuclear stain, blue). Scale bar, 50 μm. (**b**) Total DNA and protein levels increased over the time course of the experiment (*n* = 3–4 muscle strips per time point). There was no statistically significant difference in protein/DNA ratios between days 8 (17 ± 2.8 μg protein μg DNA^−1^) and 12 (15.7 ± 2.5 μg protein μg DNA^−1^). (**c**) Muscle creatine kinase (MCK) activity was significantly increased as early as day 6 and reached a maximum output on day 12 (*n* = 3 muscle strips per time point). The rate of increased MCK activity slowed between days 8 and 12, confirming that this was a relevant stopping point for the experiments. All plots represent mean ± SEM. * indicates significance (*p* < 0.05) between conditions at the same time point; ^ indicates significance compared to initial time point.

C2C12 myoblasts at a density of 5 × 10^6^ cells ml^−1^ were mixed with ECM proteins including fibrinogen, thrombin, and Matrigel^TM^; the liquid suspension was added to a 3D printed holder and began to cross-link into a gel. The cells applied traction forces to compact the tissue into a solid muscle strip around the pillars of the hydrogel skeleton. After three days in proliferation media, the bio-bot (consisting of the hydrogel skeleton and attached muscle strip) was released as a freestanding biological machine. Myoblasts were then induced to differentiate with the addition of horse serum and insulin-like growth factor (IGF-1)^31^. Striated, multinucleated myotubes were visible throughout the muscle strip (Fig. 1a).

Over the course of the experiment, the total DNA present in the muscle strips increased over ten-fold, from 2.1 ± 0.8 μg on day 3 to 23.8 ± 3.4 μg on day 12. The total protein content per muscle strip increased as well and was significantly amplified on day 12 (356.8 ± 33.5 μg) compared to day 3 (235.7 ± 44.7 μg; Fig. 1b). The activity of muscle creatine kinase (MCK), which is expressed in mature skeletal muscle only after differentiation^32^ and thus served as a quantitative gauge of myogenesis, was significantly increased as early as day 6 and reached a maximum output of 560.4 ± 44 U L^−1^ on day 12 compared to 4.2 ± 0.2 U L^−1^ on day 3 (Fig. 1c).

### Cathepsin and MMP Zymography without ACA

**Figure 2:**
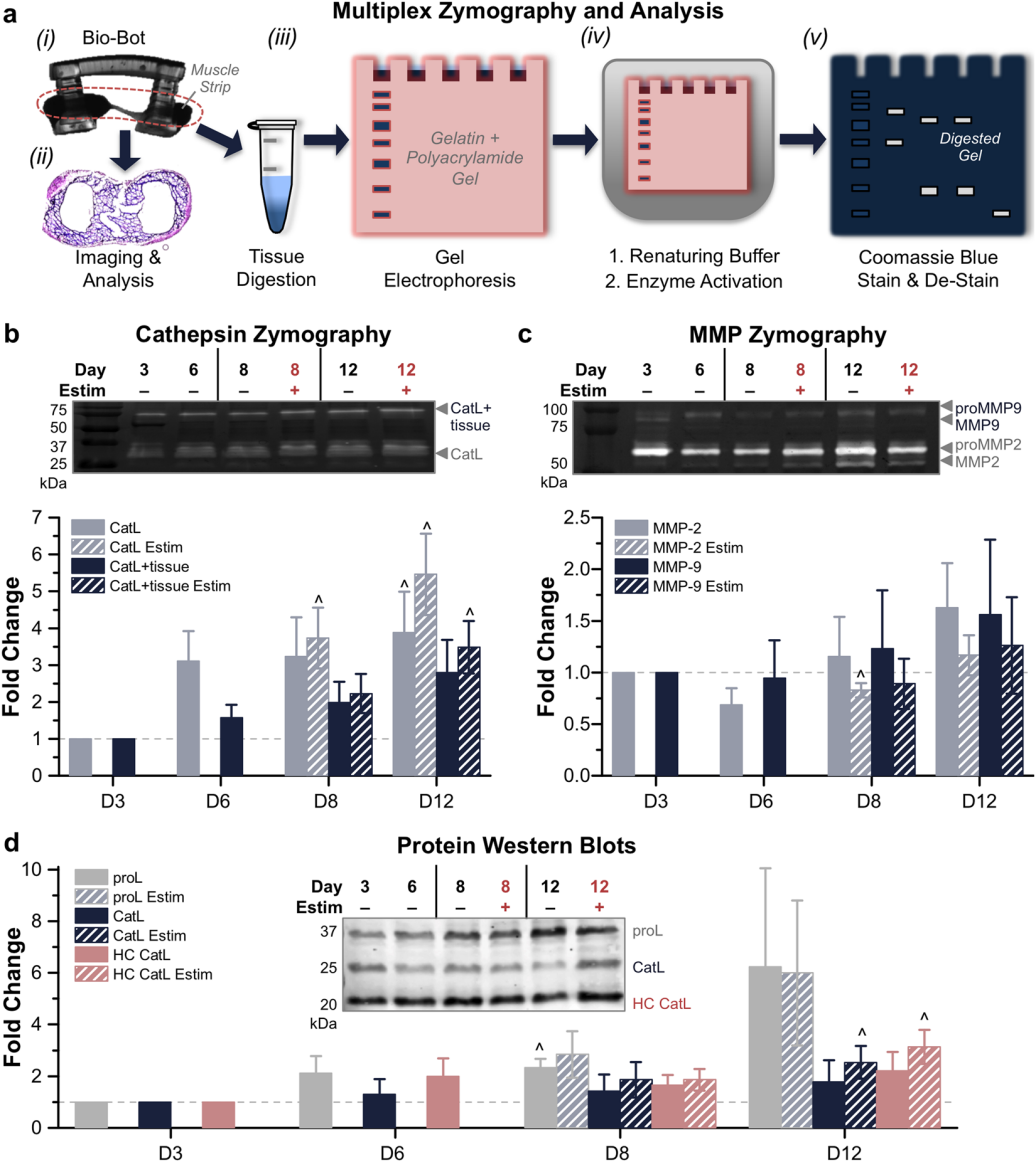
Gelatin Zymography of Cathepsins and MMPs without ACA. (**a**) Muscle strip zymography process flow. *(i)* Degrading muscle strips were removed from hydrogel skeletons. *(ii)* Muscle strips could be fixed and stained for further analysis. *(iii)* Tissues were digested in lysis buffer. Isolated proteins were run through gel electrophoresis. *(iv)* Gels were incubated in renaturing and assay buffers. *(v)* Gels were stained with Coomassie Blue; bright bands indicated the activity of specific proteases, which were separated by molecular weight. (**b**) Cathepsin zymography identified the activity of CatL and CatL + tissue at days 3, 6, 8 and 12, in the presence or absence of electrical stimulation (*n* = 3–5 muscle strips per condition). (**c**) MMP zymography identified the activity of MMP-2 and MMP-9 at days 3, 6, 8 and 12, in the presence or absence of electrical stimulation (*n* = 3–5 muscle strips per condition). For MMP zymograms, upper bands reflect inactive enzyme (proMMP) and lower bands reflect active enzyme. (**d**) Western blotting confirmed the presence of cathepsins and related proteins, such as CysC (*n* = 3–5 muscle strips per condition). All plots represent mean ± SEM. ^ indicates significance (*p* < 0.05) compared to initial time point.

Multiplex zymography was used to examine the activity of cell-secreted or cell-associated cysteine cathepsins and matrix metalloproteinases in muscle strips at various time points (Fig. 2a). Polyacrylamide gels were impregnated with gelatin to create a substrate that could be degraded by active proteases. Muscle strips were lysed and loaded for zymography. After protein separation by electrophoresis, gels were washed in a renaturing buffer that allowed proteins to re-fold into their native conformation and then incubated in assay buffer overnight for optimal enzyme activity to degrade the substrate. Both renaturing and assay buffers were specific to the proteases examined. Gels were stained with Coomassie Blue stain, which identifies all proteins and then destained; areas of white bands indicated proteolytic activity. On the cathepsin zymogram, bands were identified for Cathepsin L, both unbound (CatL, 25–37 kDa) and bound to matrix proteins in the muscle strip tissue (CatL + tissue, 75 kDa; Fig. 2b). On the MMP zymogram, bands were identified at 62–72 and 82–92 kDa, pertaining to MMP-2/pro-MMP-2 and MMP-9/pro-MMP-9, respectively (Fig. 2c).

In the absence of any anti-fibrinolytic treatment (0x ACA), muscle strips produced active cathepsins and MMPs and the amount of active enzyme increased over the course of 12 days. The activity of each protease was quantified by densitometry and normalized to the beginning of skeletal muscle differentiation on day 3. On day 12, the amount of active CatL displayed a significant (3.9 ± 1.1-fold) change and amount of active CatL + tissue increased by 2.8 ± 0.9-fold. Electrical stimulation of muscle strips significantly increased the expression of both active CatL and CatL + tissue (5.5 ± 1.1 and 3.5 ± 0.7-fold compared to day 3, respectively; Fig. 2b). Additionally, the amount of active MMP-2 and MMP-9 in muscle strips both increased on day 12, both with (1.2 ± 0.2 and 1.3 ± 0.5-fold) and without electrical stimulation (1.6 ± 0.4 and 1.6 ± 0.7-fold; Fig. 2c). Western blotting confirmed the identity of two mature isoforms of the cysteine protease CatL (including heavy chain [HC] CatL), as well as the presence of cystatin C (CysC), an endogenous cysteine protease inhibitor. Pro-forms of both MMPs and CatL were also present (Fig. 2c,d).

### Muscle Strip Life Expectancy

**Figure 3:**
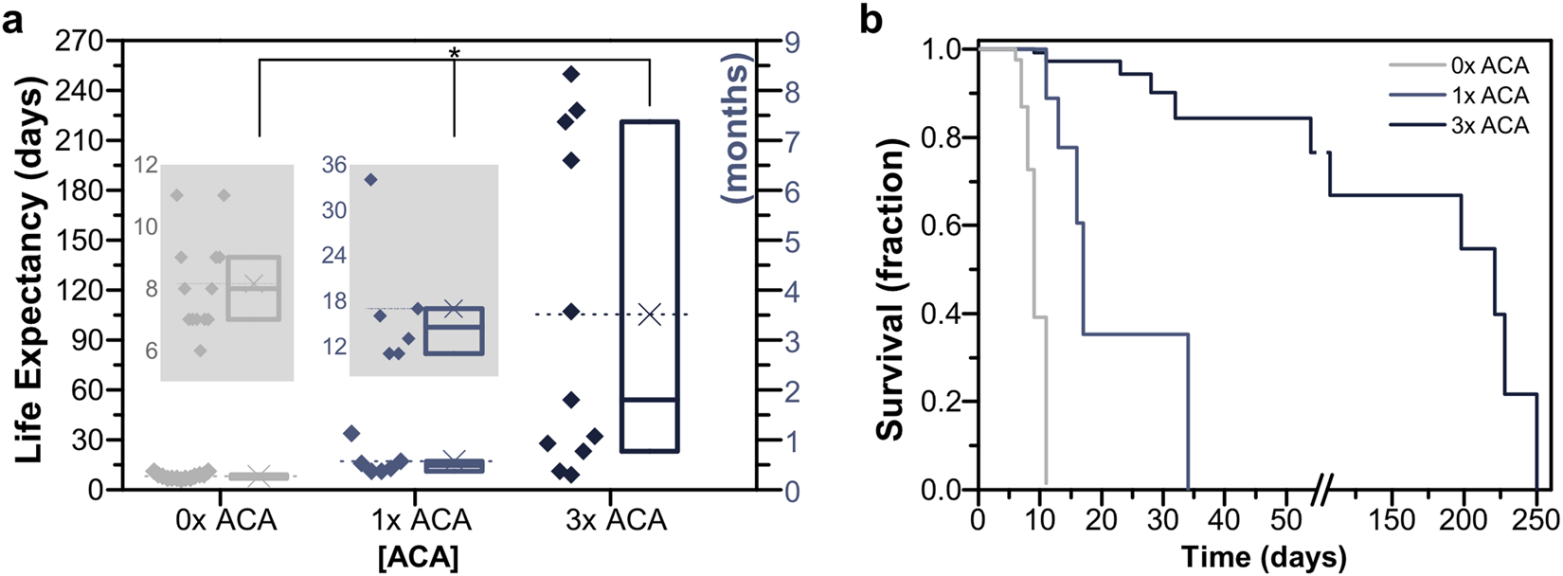
Bio-Bot Life Expectancy. (**a**) In the absence of any anti-fibrinolytic treatment (0x ACA), muscle strips demonstrated an average life expectancy of 8.2 ± 0.5 days until rupture. However, incubation in medium with the serine protease inhibitor ACA lengthened the life expectancy of the muscle strips. Box plots represent 25^th^, 50^th^ and 75^th^ percentiles, with average values marked as (x) and whiskers representing ± SEM. Data are presented to the left of the boxes (*n* = 6–13 muscle strips per condition, excluding outliers). * indicates significance (*p* < 0.05) between time points. (**b**) Kaplan-Meier survival analysis provided an additional method of comparison by plotting survival fraction of each treatment group as a function of time.

Breakdown of the muscle strip was defined as rupture or detachment from the hydrogel skeleton (Fig. 2a), rendering the bio-bot non-functional. In the absence of any anti-fibrinolytic treatment (0x ACA), muscle strips demonstrated an average life expectancy of 8.2 ± 0.5 days until rupture (Fig. 3a). However, incubation of the serine protease inhibitor ACA in medium lengthened the life expectancy of the muscle strips; addition of 1 mg ml^−1^ (1x) and 3 mg ml^−1^ (3x) ACA significantly increased the averages to 17 ± 3.9 and 105.5 ± 31.1 days until rupture, respectively. Kaplan-Meier survival analysis provided an additional method of comparison between treatment groups (Fig. 3b and Supplementary Fig. S3a). For those muscle strips which remained unruptured on the order of months, passive tension remained statistically unchanged on day 140 (4.6 months; Supplementary Fig. S3b) compared to early values; furthermore, some muscle strips treated with 3x ACA continued to contract spontaneously and in response to electrical stimulation after 210 days (7 months; Supplementary Fig. S3c and Supplementary Videos 1 and 2). Finally, increasing the concentration of ACA did not significantly alter cellular viability in the muscle strips (126.6 ± 15% for 1x ACA and 139 ± 7.8% for 3x ACA on day 12 normalized to day 3; Supplementary Fig. S4a).

### Loss of Tissue Structure and Mechanical Integrity

**Figure 4:**
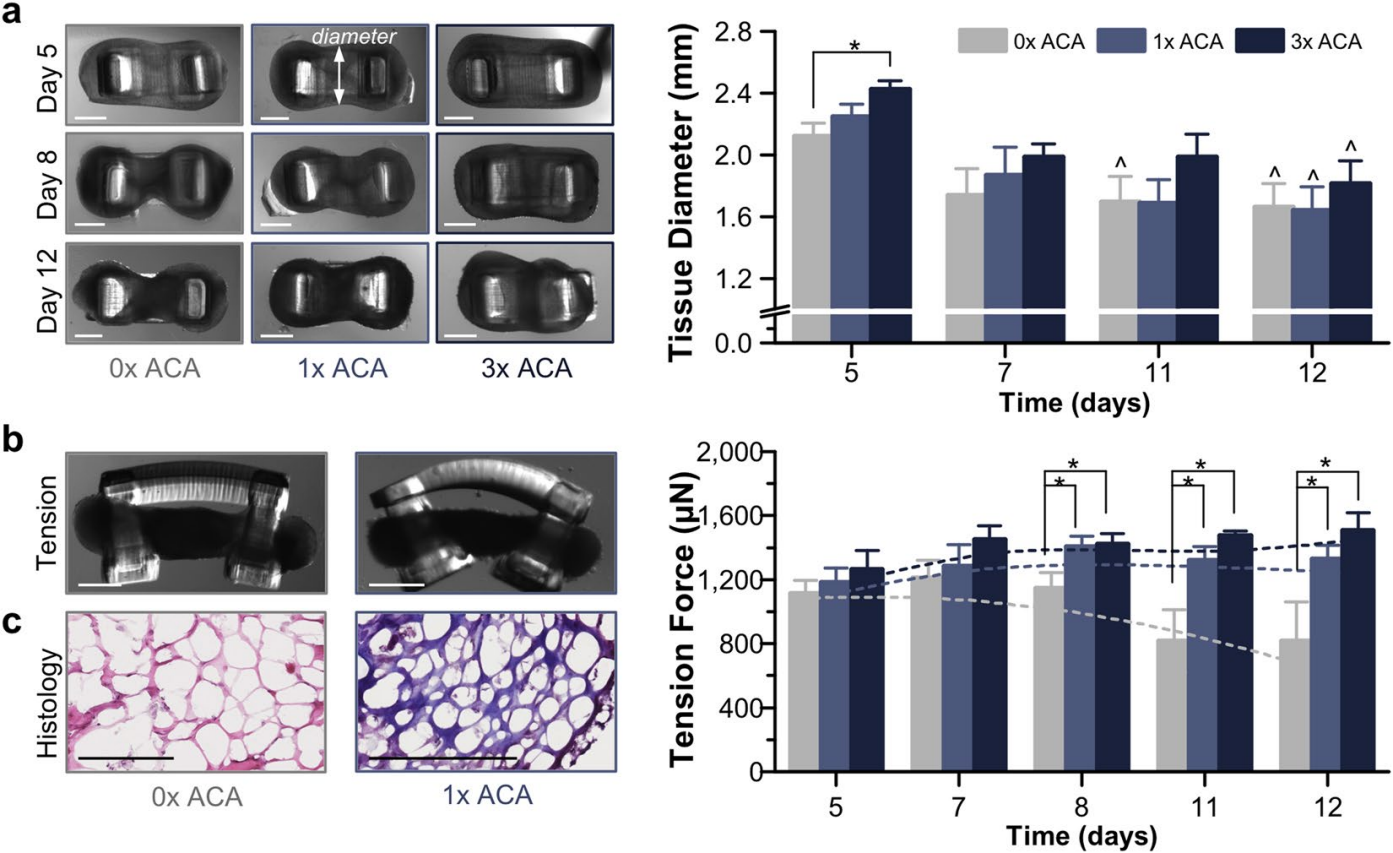
Loss of Tissue Structure and Mechanical Integrity. (**a**) Muscle strips began to degrade (most notably, in the middle region of the tissue), as shown in top-view images. Though the diameters of all groups on day 12 were reduced, the addition of 3x ACA helped to maintain the integrity of the tissue longer (*n* = 2–9 muscle strips per condition). Scale bar, 1 mm. (**b**) Muscle strips cultured without ACA displayed a lower passive tension and faster decrease in static force over time. After day 8, the passive tension was significantly lower for 0x muscle strips (*n* = 4–7 per time point) compared to both 1x (*n* = 5–6 per time point) and 3x ACA (*n* = 5–7 per time point). Scale bar, 1 mm. All plots represent mean ± SEM. * indicates significance (*p* < 0.05) between conditions at the same time point; ^ indicates significance compared to initial time point. (**c**) Histological staining for Masson’s Trichrome indicated that the addition of ACA helped to maintain the ECM in muscle strips. Scale bar, 200 μm.

Over the time course of the experiment, muscle strips began to degrade (most notably, in the middle region of the tissue) in the absence of anti-fibrinolytic treatment (Fig. 4a). Five days after cell seeding, 0x ACA muscle strips displayed cross-sectional diameters already significantly reduced from those treated with the inhibitor (2.1 ± 0.08 versus 2.4 ± 0.06 mm for 3x ACA). On day 12, the diameters of all groups were significantly reduced (a range of 73.1 ± 6.8 to 78.4 ± 7% from their day 5 values; Supplementary Fig. S4b). The 0x ACA muscle strips displayed a lower passive tension and faster decrease in static force over time compared to both 1x and 3x ACA treated muscle strips. As early as day 8, the passive tension was significantly lower for 0x ACA; by day 12, it had dropped to 815.5 ± 247.1 μN, compared to 1327.6 ± 86 and 1509.5 ± 106.7 μN for 1x and 3x ACA, respectively (Fig. 4b). Neither application of daily electrical stimulation nor increasing the concentration of ACA beyond 1x caused any statistical difference in average passive tension during this time span (Supplementary Fig. S4c). Additionally, histological staining for Masson’s Trichrome, a connective tissue stain, indicated that the addition of ACA helped to maintain the presence of ECM proteins supporting the differentiated myotubes (Fig. 4c).

### Cathepsin and MMP Zymography with 1x and 3x ACA

**Figure 5:**
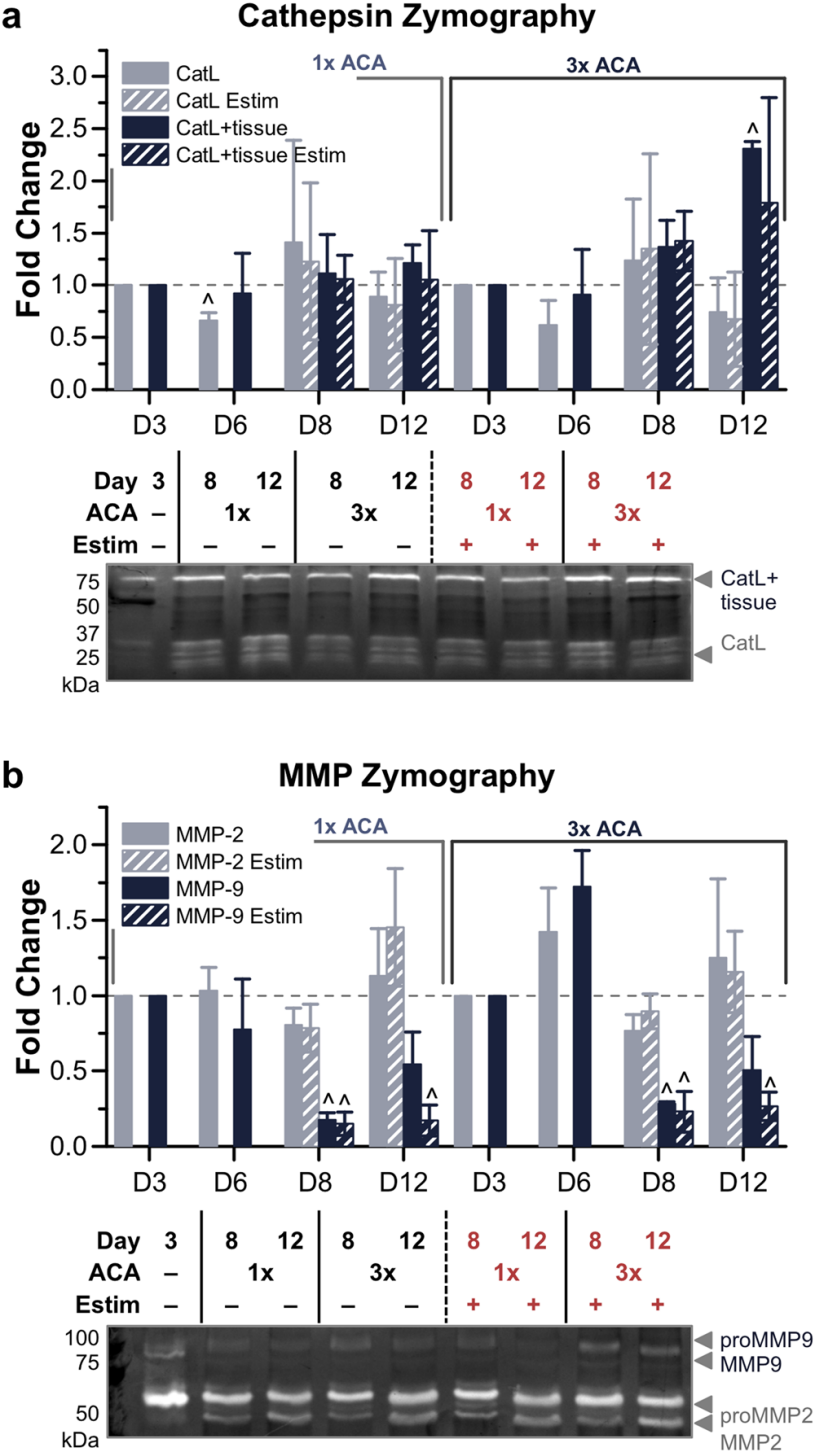
Gelatin Zymography of Cathepsins and MMPs with 1x and 3x ACA. (**a**) Cathepsin zymography identified the amount of active CatL and CatL + tissue at days 3, 6, 8 and 12, in the presence or absence of electrical stimulation, for 1x and 3x ACA (*n* = 3–6 muscle strips per condition). (**b**) MMP zymography identified the amount of active MMP-2 and MMP-9 at days 3, 6, 8 and 12, in the presence or absence of electrical stimulation, for 1x and 3x ACA (*n* = 4–7 muscle strips per condition). All plots represent mean ± SEM. ^ indicates significance (*p* < 0.05) compared to initial time point.

In the presence of a serine protease inhibitor, muscle strips cultured with 1x and 3x ACA continued to produce active cathepsins and MMPs. On day 12, the amount of active CatL displayed a 0.9 ± 0.2-fold change with 1x ACA and a 0.7 ± 0.3-fold change with 3x ACA compared to day 3. The amount of active CatL + tissue displayed a 1.2 ± 0.2-fold change with 1x ACA and a 2.3 ± 0.1-fold change with 3x ACA, significantly increased from day 3 (Fig. 5a). Both the amount of active MMP-2 and MMP-9 increased by day 6, but decreased below day 3 levels on day 8. On day 12, MMP-2 was again upregulated (1.1 ± 0.3-fold and 1.3 ± 0.5-fold change for muscle strips cultured with 1x and 3x ACA), while levels of active MMP-9 continued to decrease significantly compared to day 3 levels (0.5 ± 0.2-fold and 0.5 ± 0.2-fold change for muscle strips cultured with 1x and 3x ACA; Fig. 5b).

**Figure 6:**
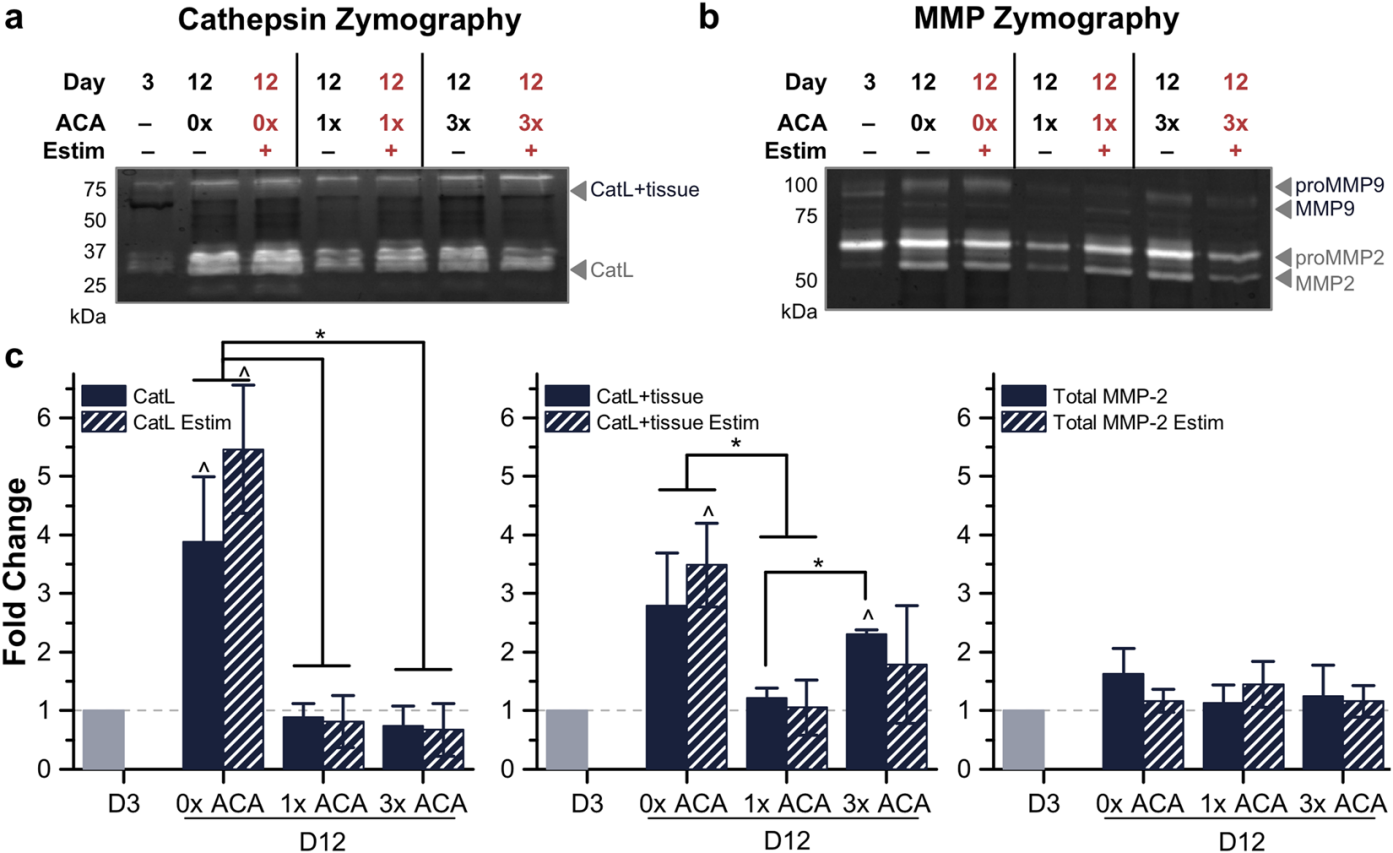
Comparison of Active Cathepsins and MMPs in 0x, 1x and 3x ACA Cultured Muscle Strips. (**a**) Cathepsin zymography identified the amount of active CatL and CatL + tissue and (**b**) MMP zymography identified the amount of active MMP-2 and MMP-9, with and without electrical stimulation, for day 12 conditions compared to the initial time point of day 3. (**c**) ACA prolonged the lifetime and reduced the amount of active CatL in locomoting biological machines. All plots represent mean ± SEM. * indicates significance (*p* < 0.05) between conditions at the same time point; ^ indicates significance compared to initial time point.

However, when comparing muscle strips cultured with 0x ACA to those cultured in 1x and 3x ACA after 12 days, we observed a reduction in the amount of active protease that was statistically significant for CatL (0.9 ± 0.2-fold and 0.7 ± 0.3-fold, compared to 3.9 ± 1.1-fold for 0x), but not significant for MMP-2 or MMP-9 (Fig. 6). Increasing the ACA concentration beyond 1x did not further enhance this reduction in active CatL and electrical stimulation did not significantly alter the expression of any of the cathepsins or MMPs cultured with ACA. Finally, Western blotting also verified the presence of the pro-form of CatL, as well as the presence of CysC (Supplementary Fig. S5).

### Varying Hydrogel Skeleton Stiffness and Cell Seeding Density

**Figure 7:**
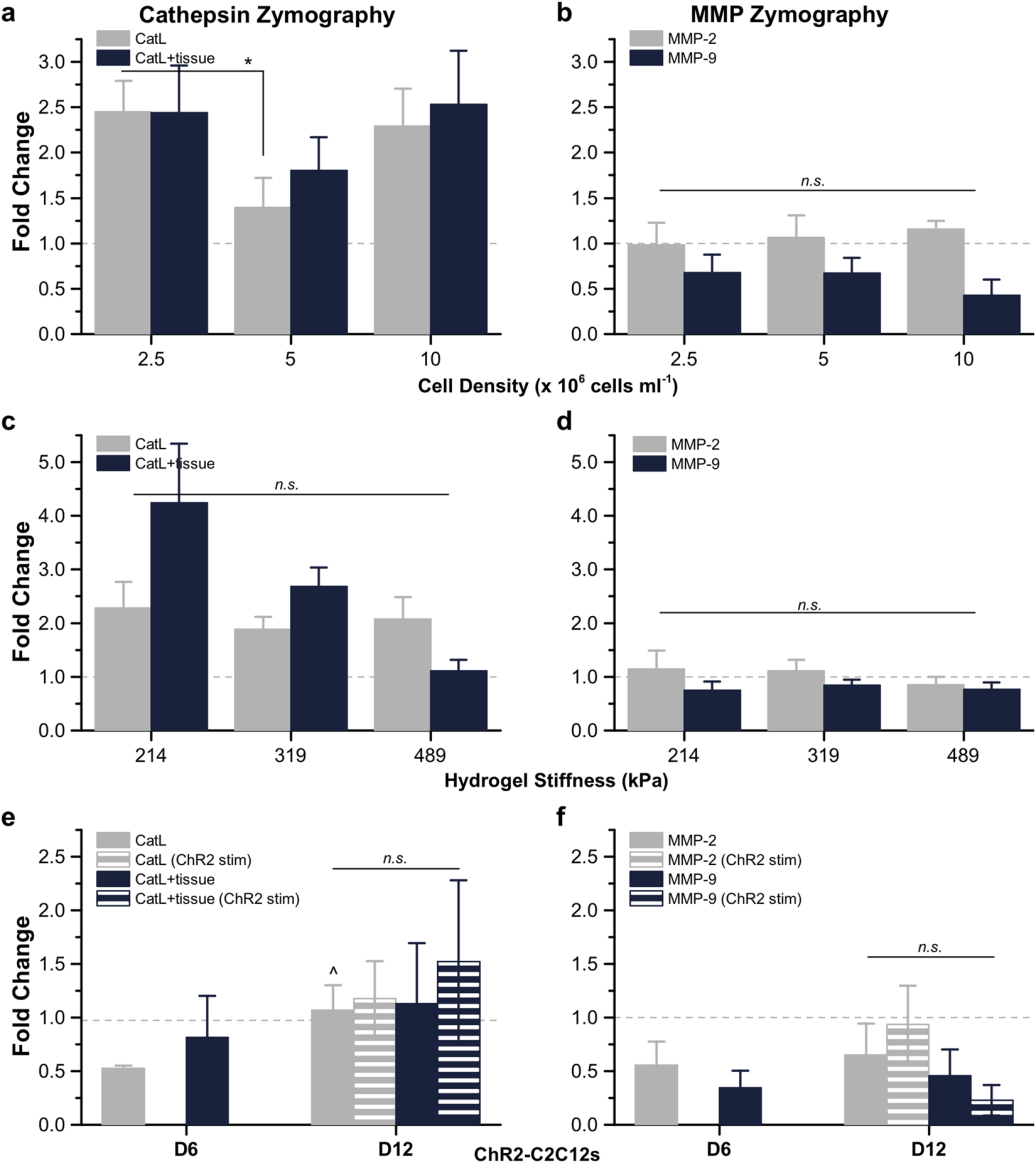
Gelatin Zymography of Cathepsins and MMPs in Muscle Strips with Varying Cell Density, Hydrogel Skeleton Stiffness and Applied Stimulation. (**a**) For day 12 muscle strips cultured with 1x ACA and fabricated with varying cell densities (2.5, 5, or 10 × 10^6^ cells ml^−1^), cathepsin zymography identified the amount of active CatL and CatL + tissue and (**b**) MMP zymography identified the amount of active MMP-2 and MMP-9 (*n* = 7−10 muscle strips per condition). (**c**) For day 12 muscle strips cultured with 1x ACA with a hydrogel skeleton stiffness of 214, 319, or 489 kPa, cathepsin zymography identified the amount of active CatL and CatL + tissue and (**d**) MMP zymography identified the amount of active MMP-2 and MMP-9 (*n* = 9−17 muscle strips per condition). (**e**) For ChR2-C2C12 optogenetic muscle strips, cathepsin zymography identified the amount of active CatL and CatL + tissue at days 6 and 12 and (**f**) MMP zymography identified the amount of active MMP-2 and MMP-9 at days 6 and 12, in the presence or absence of optogenetic stimulation (*n* = 3 muscle strips per condition). All plots represent mean ± SEM. * indicates significance (*p* < 0.05) between conditions at the same time point; ^ indicates significance compared to initial time point (day 6 for panels (e and f).

The hydrogel-muscle platform was modified to introduce tunable variations in the muscle strip as well as the 3D printed skeleton^4^. First, to test the hypothesis that concentration of cells within the muscle strip affected the amount of active proteases produced, muscle strips seeded at a density of 5 × 10^6^ cells ml^−1^ were compared to those with 2.5 and 10 × 10^6^ cells ml^−1^. On day 12, though a decreased cell density caused a significant increase in CatL, other measurements of active CatL and CatL + tissue showed no significant change compared to muscle strips with 2.5 or 10 × 10^6^ cells ml^−1^ cultured in 1x ACA (Fig. 7a and Supplementary Fig. S6a). Likewise, MMP-2 and MMP-9 also showed no significant change compared to muscle strips with 2.5 or 10 × 10^6^ cells ml^−1^ (Fig. 7b and Supplementary Fig. S6a).

Next, to assess whether cells in the muscle strip responded differently when subjected to a varying degree of static force, we altered the mechanical microenvironment felt by the myotubes during differentiation by 3D printing hydrogel skeletons with varying stiffness values (see Methods). When cultured in the serine protease inhibitor ACA, muscle strips coupled to hydrogel beams with a modulus of 319 kPa (an optimal stiffness, used in previous bio-bot demonstrations, that combined a robust mechanical structure with sufficiently high deflection necessary for locomotion^4, 5^) had retained their passive tension over time (Fig. 4c). Muscle strips cultured in 1x ACA for 12 days in higher tension (489 kPa beam modulus) or lower tension (214 kPa beam modulus) showed no significant change in the amount of active CatL or CatL + tissue compared to those on skeletons with a beam modulus of 319 kPa (Fig. 7c and Supplementary Fig. S6b). Though MMP-2 showed a downward trend with increasing tension, there was no significant change for either MMP-2 or MMP-9 in an environment of higher or lower tension (Fig. 7d). Western blotting corroborated the presence of the pro-form of CatL as well as CysC in these muscle strips (Supplementary Fig. S6c).

### Optical Stimulation of ChR2-C2C12 Muscle Strips

To test if dynamic optical stimulation of muscle strips contributed to an increase in protease activity, bio-bots were fabricated using ChR2-C2C12s (myoblasts infected to express an optogenetic ion channel^5^). ChR2-C2C12 optogenetic bio-bots were cultured in 1x ACA and subjected to daily optical stimulation by an external blue light pulse that depolarized the cells to produce contraction. The muscle strips from ChR2-C2C12 optogenetic bio-bots were collected on day 6 (to compare to non-optogenetic bio-bots) and on day 12 (to compare to both non-optogenetic bio-bots as well as ChR2-C2C12 bio-bots without optogenetic stimulation) for zymography. Just as daily electrical stimulation had not altered the expression of CatL or MMP-2 over 12 days, the application of daily optical stimulation for ChR2-C2C12 optogenetic bio-bots did not have a significant impact on the amount of active CatL, CatL + tissue, MMP-2, or MMP-9 (Fig. 7e,f and Supplementary Fig. S7a,b). Finally, there was no fundamental difference between ChR2-C2C12 and non-optogenetic muscle strips with regards to the amount of active cathepsins or MMPs at either time point and optical stimulation of ChR2-C2C12 optogenetic bio-bots resulted in similar trends in active CatL, CatL + tissue, MMP-2, and MMP-9 expression as electrical stimulation (Supplementary Fig. S7c-f). Western blotting confirmed the presence for pro-form of CatL and CysC in the ChR2-C2C12 optogenetic bio-bots (Supplementary Fig. S7g).

## Discussion

The role of the basal lamina is of critical importance in providing necessary elasticity and strength, as well as key binding sites for sequestration of myogenic growth factors, during muscle development. Laminin-2, collagen IV, and proteoglycans are present in abundance in skeletal basal lamina. In the hydrogel-muscle platform of the skeletal muscle bio-bots, natural ECM proteins comprise a bioinspired engineered system that provides mechanical and biochemical support to differentiating myotubes in a physiological arrangement. After myoblasts withdraw from the cell growth cycle and commit to a myogenic lineage during differentiation, they migrate to (and then physically fuse with) other myoblasts to create multinucleated myotubes with one cytoplasm and increase expression of muscle-specific genes in adult muscle fibers^16, 31, 33^. The cells in the muscle strips exhibited these morphological and genetic changes, observable by immunostaining (Fig. 1a) and the presence of mature muscle proteins (Fig. 1c). During injury, a similar process of migration and fusion is observed by quiescent satellite cells, or myoblast progenitors, present in the basal lamina^34^.

*In vivo*, protease activity is regulated by a strict balance between expression, activation, and inhibition; this balance plays a major role in the degree of matrix degradation and resulting cellular processes^24^. *In vitro*, degradation and resulting instability are drawbacks to tissue engineering with fibrin and an inhibitor such as the anti-fibrinolytic agent ACA^13^ must be incorporated to prevent indiscriminate matrix digestion in systems lasting longer than a few hours. Incubation with increasing concentrations of ACA helped to preserve the structural integrity of the fibrin muscle strips and significantly increased the lifetime before rupture (Fig. 3). Though the serine protease inhibitor aided in the maintenance of passive tension in the muscle strips over time and slowed the degradation of muscle strips at earlier time points, it did not significantly reduce tissue loss by day 12 (Fig. 4); thus, the need for an investigation of the other protease families, including cysteine cathepsins and MMPs, was apparent.

*In gel* zymography is a versatile method used to determine protease activity by incubation in a gel substrate composed of degradable proteins. As it does not require antibodies, is relatively inexpensive and allows for visual confirmation of enzyme identity (and determination of active quantity) by molecular weight separation, zymography is useful in the study of both cathepsins and MMPs^35, 36^ across a number of tissue types^37–40^. Using a well-characterized technique wherein electrophoresis separated proteases of interest from muscle strip lysate^35, 36^, we temporally quantified the amount of active cathepsins as well as MMPs in various culture conditions (Figs 2b,c, 5, 6, and 7). Cathepsins are implicated in a number of tissue-destructive disease states as well as mechanisms of normal tissue physiology, including myogenic differentiation^35, 38, 41–44^. CatL has been recognized as a major player in muscle atrophy and wasting^16, 33^. We observed that the amount of active CatL present in the muscle strips increased steadily over time and was most highly expressed in the absence of ACA. Its activity on day 12 was significantly higher in all 0x muscle strips compared to 1x and 3x ACA, both with and without electrical stimulation (Fig. 6c), indicating that the presence of ACA may prevent some processes associated with physiological muscular breakdown and potentially reduce the expression of CatL. There is also prior evidence suggesting ACA may inhibit proteolytic activity by lysosomal cysteine cathepsins^45, 46^. These hypotheses could explain the extended lifetime observed in the presence of ACA and why this process is independent of electrical stimulation.

MMPs comprise a range of proteases capable of degrading all ECM proteins. Like cathepsins, they are also involved in many normal mammalian cell processes (such as migration, adhesion, and proliferation) and their upregulation has been reported in various pathologies. Their role in myogenic differentiation, development, and injury is primarily extracellular and they have been shown to interact with specific ECM proteins over varied timelines^47^. Cellular alignment, differentiation of adult myofibers, or remodeling of muscle tissue architecture is a direct result of MMPs degrading ECM proteins, which may hinder myoblast migration and fusion^18, 34^. MMPs can also be activated (directly or indirectly) by other proteolytic enzymes including plasmin and plasminogen^25^, which are present in our system. Both MMP-2 and MMP-9 can act on various collagen substrates, some of which are localized in our engineered muscle strips due to the presence of Matrigel^TM^ ^18^.

Expression of MMP-9 has been shown to be upregulated in C2C12 myoblast cultures during migration and before cell fusion^17, 34^. The processes concerning mature myotube formation then witness a decrease in expression over time. We observed a decline in amount of active MMP-9 at later time points in muscle strips cultured with 1x and 3x ACA (Figs 5b, 7f, and Supplementary Fig. 7d,f). Since upregulation of MMP-9 is also associated with early acute inflammation and initiation of muscle regeneration in proliferating and migrating satellite cells^34^, it is likely that only the earlier stages of muscle strip compaction and differentiation involve processes that resemble the recruitment (and subsequent fusion) of satellite myoblasts after injury.

MMP-2, which is secreted by C2C12s, is constitutively produced in lower levels in healthy adult muscle and does not play a large role in myoblast migration or fusion. It is involved more heavily in later stages of C2C12 myofiber differentiation^17, 24, 34^. Active MMP-2 was slightly increased in all conditions on day 12 compared to day 3 (Figs 5b and 6c), suggesting that while myogenic differentiation was observed, the complete extracellular remodeling of matrix proteins that accompany later stages of muscular maturation may not have been visible during this experimental timeline. Though a short-term investigation proved relevant for this application of biological machines, an investigation on a longer time scale would provide more detailed temporal data on expression of specific MMPs as well as cathepsins. Additionally, levels of MMP-2 (which can be secreted by satellite cells) increase gradually during later stages of injury, then return to baseline levels, suggesting an important role in repair and regeneration of new muscle fibers^17, 24^. The introduction of deliberate inflammation or injury to the muscle strips could test this hypothesis in future studies.

The fabrication of skeletal muscle bio-bots offers flexibility in varying almost any parameter in the system. We also investigated the effect of altering certain variables whose affects on bio-bot functionality we have previously reported: cell density, hydrogel stiffness^4^ and dynamic optogenetic stimulation^5^. Varying these conditions revealed that the amount of active cathepsin and MMP was largely independent of these environmental changes or applied stimuli. When the cell seeding density for muscle strip formation was varied, the amount of active cathepsin and MMP was statistically unchanged (Fig. 7a,b and Supplementary Fig. 6a). MMP-2 increased slightly, though not significantly, as the cell concentration was increased to 10 × 10^6^ cells ml^−1^; it is possible that the increase in initial myoblast density accelerated the fusion and differentiation process. MMP-2 has been shown to increase with the presence of mature myotubes^24, 25^, substantiating this observation.

When muscle strips were differentiated under higher static tension, the amount of active cathepsin L decreased, though again not significantly (Fig. 7c and Supplementary Fig. 6b,c). Interestingly, the amount of active MMP-2 and MMP-9 both decreased in the presence of higher static tension (Fig. 7d), despite an extracellular presence in a condition that favors higher degrees of myofiber alignment and differentiation. This may indicate reduced myotube formation in these bio-bots under higher tension.

Finally, when ChR2-C2C12 optogenetic bio-bots were subjected to optical stimulation, we observed no change in the amount of active CatL or MMPs (Fig. 7e,f and Supplementary Fig. 7). Though electrical stimulation is a useful tool because it allows for the global and coordinated stimulation of all excitable cells within the muscle strip, optical stimulation provides a less invasive and more specific alternative by increasing spatiotemporal control over contraction and locomotion^5, 48^. These results indicate that neither the use of optogenetic cells nor optical stimulation altered proteolytic expression profiles associated with myogenic differentiation or injury and confirm that the electrical stimulation is not responsible for the observed proteolytic activity by cathepsins and MMPs.

In engineered living systems, biological building blocks – different cell types or tissues in an instructive environment – can be assembled to promote the emergence of cellular systems with well-defined functionality, allowing for the realization of dynamic cellular machines with the ability to interface with the environment and other living systems^1^. However, as these biological machines increase in complexity, it may be critical to design systems with a specific life expectancy and exhibit greater consistency. Forward engineering of biological and mechanical properties, along with knowledge of how these factors affect protease activity, can dictate a system’s sustainability. A machine designed to ‘fail’ or ‘break’ within a certain time frame (after performing a specific task, for example) could be programmed to upregulate expression of proteases through inflammatory cytokines such as TNF-α, which are known to accelerate overexpression or activation of some proteases^17, 42^. On the other hand, the addition of a protease inhibitor (such as the strong-binding endogenous cysC^35, 49, 50^, the broad-spectrum cathepsin inhibitor E-64^35, 42, 50^, or tissue inhibitors of metalloproteinases [TIMPs]^18, 51^) could aid in prolonging the machine’s lifespan by lowering the amount of active proteases. Most likely, the majority of future engineered living systems will require an intricate balance between the activity and inhibition of serine and cysteine proteases as they exist and function together in a complex, inter-connected proteolytic network^52, 53^. The ratio between proteases and their inhibitors (such as the important equilibrium required between MMPs and TIMPs in skeletal muscle^17, 18, 25, 34^) is critical to maintaining a balanced state.

Looking forward, as neuromuscular control and vascularization are introduced into engineered living systems, it will be critical that we understand what proteases are involved in these processes and how the cells may adapt to the new environment. Proteolysis of the basal lamina is critical in angiogenesis and MMP-2 and membrane-type 1 (MT1) MMP involvement has been seen in capillary growth^17^. Furthermore, MMP-2, MMP-7 and MMP-9 are co-localized at neuromuscular junctions in healthy tissue^17^. Taken together, these highlight the importance of recognizing that the proteases that appear to be involved in normal muscle strip development will also be involved in these other cellular processes and must be accounted for in future development of engineered living systems.

Finally, because skeletal muscle cathepsins can also hydrolyze myogenic proteins such as myosin, troponin T and tropomyosin^33, 54^, it may also be worth examining whether the activity of these proteases in intracellular degradation (and subsequent breakdown or atrophy of muscle fibers) contributes in any way to failure of the engineered muscle system. Lastly, increased expression and regulation of cathepsins^33, 55^ and MMPs^18^ has been observed in dystrophic muscles; therefore this platform could also be used for the study of disease-specific models and treatments of diseases such as muscular dystrophy or other myopathies.

## Methods

### Design and 3D Printing of Bio-Bot Skeletons

Bio-bot skeletons and holders were fabricated using a commercial Stereolithography apparatus (SLA 250/50, 3D Systems)^4^. Briefly, parts were designed in CAD software (AutoCAD), exported in. STL format for slicing into layers (3D Lightyear software, v1.4, 3D Systems) and built in a layer-by-layer fashion on a modified SLA stage^56, 57^. To print hydrogel beams with varying stiffness profiles, the polymerization energy dose of the SLA (108.7 to 233.3 mJ cm^−2^) was adjusted according to a Working Curve Equation^29, 30^ in order to obtain elastic moduli of 214 to 489 kPa, as detailed earlier^4^. Liquid hydrogel formulations were not altered. A skeleton beam of 319 kPa was used unless otherwise noted.

Liquid pre-polymer hydrogel solutions were composed of 20% poly(ethylene glycol) diacrylate of M_W_ 700 g mol^−1^ (PEGDA 700, Sigma-Aldrich) or poly(ethylene glycol) dimethacrylate of M_W_ 1000 g mol^−1^ (PEGDMA 1000, Polysciences, Inc.)^4, 5^. After fabrication, skeletons and holders were sterilized in 70% EtOH and stored in sterile PBS.

### Cell Culture and Seeding

C2C12 murine myoblasts were proliferated in growth medium (GM) and passaged before confluence. ChR2-C2C12s were used for optogenetic bio-bots, as previously described^5, 58^. GM consisted of Dulbecco’s Modified Eagle Medium (DMEM, Corning Cellgro) with 10% (v/v) fetal bovine serum (FBS, VWR) and 1% (v/v) each of L-glutamine and penicillin-streptomycin (both Corning Cellgro). For the seeding of muscle strips, bio-bot skeletons were placed in polymerized holders and aspirated of excess liquid. 5 × 10^6^ cells ml^−1^ (final concentration of myoblasts in cell-gel solution, unless otherwise noted) were resuspended in GM and mixed with ice-cold Matrigel^TM^ basement membrane (30% of total volume, Corning), fibrinogen (4 mg ml^−1^, Sigma-Aldrich) and thrombin from bovine plasma (0.5 U mg^−1^ fibrinogen, Sigma-Aldrich). Cell-gel solution was added to each holder (day 0) and incubated for 1.5 hr before adding warm GM. Cells and cultures were maintained at 37 °C and 5% CO_2_.

### Skeletal Muscle Differentiation and Stimulation

After 3 days in GM, bio-bots were released from the holders and switched to differentiation media (DM), consisting of DMEM with 10% (v/v) heat-inactivated horse serum (HS, Gibco), 1% (v/v) each of L-glutamine and penicillin-streptomycin, 50 ng ml^−1^ of insulin-like growth factor (IGF-1, Sigma-Aldrich), and ACA as noted: 0 mg ml^−1^ (0x ACA), 1 mg ml^−1^ (1x ACA), or 3 mg ml^−1^ (3x ACA). Media was changed daily. Electrical stimulation was applied using a custom-built setup^4, 59^ starting on day 4 (Supplementary Fig. S2). Bio-bots were placed in warm DMEM between two sterilized Pt electrodes and a current was applied perpendicular to the longitudinal axis with the following parameters: 20 V amplitude, 50 ms pulse width, 1 Hz frequency, 10 min per day. Optical stimulation of ChR2-C2C12 optogenetic bio-bots was applied as previously described^5^, starting on day 4, at 1 Hz for 10 min per day.

### Force and Diameter Measurements

Bio-bot images were taken using a stereomicroscope (MZ FL III, Leica Microsystems) with a digital camera (Flex, SPOT Imaging Solutions). The ImageJ Measure Tool (National Institutes of Health) was used to measure muscle strip dimensions from top-view (diameter) and side-view (passive tension) images. Passive tension force was calculated using an equation derived from Euler-Bernoulli linear beam theory: *F*_*p*_ = (8EI/*lL* ^2^)*δ*_*max*_, where *E* is the elastic modulus (319 kPa^4^, unless otherwise noted), *I* is the moment of inertia of the bio-bot beam, *L* is the beam length, *l* is the moment arm between the beam and muscle strip, and *δ*_*max*_ is the beam deflection.

### Viability Assays

Cell viability was quantitatively determined using a metabolic colorimetric assay by incubating muscle strips in CellTiter 96 AQueous One Solution (MTS, Promega) and DMEM without phenol red (LifeTechnologies) in a 5:1 (v/v) ratio in the dark at 37 °C. After 4 h, absorbance was measured at 490 nm using a microplate reader (Synergy HT, BioTek). The absorbance of a blank sample without cells was subtracted from each reading and results were normalized to day 3 (the start of differentiation).

### Immunofluorescence and Histology

Muscle strips were removed from skeletons and rinsed in PBS. For fluorescent staining, tissues were fixed in 4% paraformaldehyde (Electron Microscopy Services) for 30 min and permeabilized with 0.25% Triton X-100 (Sigma-Aldrich) for 10 min. After blocking in Image-iT® FX (Molecular Probes) overnight at 4 °C, muscle strips were incubated with MF-20 (1:400, Developmental Studies Hybridoma Bank, The University of Iowa) and anti-sarcomeric α-actinin (1:400, Abcam) primary antibodies overnight at 4 °C, rinsed 3x with PBS and incubated with Alexa Fluor® 488 goat anti-mouse and Alexa Fluor® 568 goat anti-rabbit (both 1:400, ThermoFisher) overnight at 4 °C in the dark. Muscle strips were rinsed 3x with PBS, incubated with 4′,6-diamindino-2-phenylindole (DAPI, 1:5,000 in sterile DI water, Sigma-Aldrich) for 10 min, rinsed and imaged with a confocal microscope (LSM 710, Zeiss). For histology, frozen muscle strips were embedded in OCT compound (TissueTek), cut into 14 μm sections using a cryostat (CM3050, Leica), mounted on glass slides, stained with a Masson’s Trichrome kit (Polysciences, Inc.) and imaged using a digital pathology slide scanner (C9600, NanoZoomer).

### Muscle Strip Nucleic Acid Extraction and Assays

Muscle strips were removed from bio-bot skeletons, snap frozen in liquid nitrogen, and stored at −80 °C. Before RNA isolation, tissues were lysed using a rotor-stator homogenizer (TissueRuptor, Qiagen). Total RNA was extracted using an RNeasy Mini Kit (Qiagen). Alternatively, DNeasy Blood and Tissue Kit (Qiagen) was used to isolate total genomic DNA from muscle strips. The concentration of DNA or RNA was measured at 260 nm with a spectrophotometer (NanoDrop 1000).

### Muscle Strip Protein Extraction and Assays

Muscle strips were snap frozen in liquid nitrogen and stored at −80 °C. Upon thawing, muscle strip cells were digested in lysis buffer with freshly added 0.1 mM leupeptin (Calbiochem) for zymography and Western blotting, or RIPA buffer (Thermo Scientific) for total protein measurements and muscle creatine kinase (MCK) assays. Lysis buffer consisted of 20 nM Tris–HCl at pH 7.5, 5 mM ethylene glycol-bis(2-aminoethylether)-N,N,N′,N′-tetraacetic acid (Sigma-Aldrich), 20 mM β-glycerolphosphate (Alfa Aesar), 150 mM NaCl (BDH), 1 mM sodium orthovanadate (Sigma-Aldrich), 10 mM NaF (Sigma-Aldrich), 1% Triton X-100 (EMD Chemicals) and 0.1% Tween-20 (Fisher Scientific)^36^. After sonication and centrifugation, protein extract supernatant was collected. Using a Pierce BCA Protein Assay Kit (Thermo Scientific), total muscle strip protein content was measured at 562 nm with a spectrophotometer. Extracted protein supernatant was used to determine MCK production using a Liquid Creatine Kinase Reagent Set (Pointe Scientific) and absorbance was measured at 340 nm with a microplate reader.

### Cathepsin and MMP Zymography

Protocols for multiplex zymography to detect mature cathepsins K, L, S, and V have been previously optimized^35, 36^. MMP zymography has also been optimized for detection of pro- and mature forms of MMP-2 and MMP-9^60^. For gelatin zymography, SDS-PAGE gels (12.5% for cathepsins, 10% for MMPs) were impregnated with 5 mg ml^−1^ soluble gelatin substrate. Samples were prepared with a non-reducing loading dye (5 × −0.05% bromophenol blue, 10% SDS, 1.5 M Tris, 50% glycerol) and separated by electrophoresis resolved at 200 V for 1 h at 4 °C. Gels were washed in renaturing buffer and incubated in assay buffers overnight, specific to proteases being probed. Renaturing buffer contained 65 mM Tris buffer at pH 7.4 with 20% glycerol for cathepsins and 2.5% Triton-X 100 for MMPs. Assay buffer contained 0.1 M sodium acetate buffer at pH 4.0, 1 mM EDTA and freshly added 2 mM dithiothreitol for cathepsins and 50 mM Tris-HCl at pH 7.4, 10 mM CaCl_2_, 50 mM NaCl and 0.05% Triton X-100 for MMPs. Gels were stained for 2 h in 4.5% Coomassie blue stain (Sigma-Aldrich) with 10% acetic acid and 25% isopropanol, destained with 10% isopropanol and 10% acetic acid and imaged with an ImageQuant LAS 4000 (GE Healthcare). Densitometry quantification of white band intensity (indicating active protease) was preformed using ImageJ and results were normalized to day 3. In all results, one representative gel image is shown for each experiment.

### Western Blots

Muscle strip protein extracts were prepared with loading dye with the addition of β-mercaptoethanol, boiled for 5 min, and loaded into a 12.5% SDS-PAGE gel. Proteins were separated by molecular weight under electrophoresis resolved at 150 V and then transferred to a nitrocellulouse membrane using a Trans-Blot SD Semi-Dry Transfer Cell (Bio-Rad) at 10 V for 30 min. Membranes were blocked for >1 h in Odyssey Blocking Buffer (LI-COR Bioscience) diluted 1:2 in PBS, then incubated with primary antibodies (1:1000) for goat polyclonal mouse cathepsin L (R&D Systems), or rabbit polyclonal cystatin C (Millipore) overnight at 4 °C with agitation. Proteins were detected with a LI-COR Odyssey scanner using anti-goat or anti-rabbit secondary antibodies (1:5000 with 0.1% Tween, Invitrogen) tagged with an infrared fluorophore.

### Statistical Analysis

Results are presented as mean ± standard error of the mean (SEM). Statistical analyses included tests for significance (one-way ANOVA followed by Tukey’s Multiple Comparison Test, performed using OriginPro software, or two-tailed t-tests, performed using Microsoft Excel) and survival (Kaplan-Meier analysis, performed using OriginPro software). For muscle strip life expectancy and survival analysis, outliers were calculated using the Interquartile Range (IQR) Rule. Data points outside of 1.5 × IQR from the 1^st^ or 3^rd^ quartiles were omitted.

## Electronic supplementary material

Supplementary Information

Supplementary Video 1

Supplementary Video 2
