# Supplementary material for: Investigating the Life Expectancy and Proteolytic Degradation of Engineered Skeletal Muscle Biological Machines
Source: Sci Rep. 2017 Jun 19;7:3775. doi: 10.1038/s41598-017-03723-8 (PMC5476614; doi:10.1038/s41598-017-03723-8)
Supplement: Supplementary file 1 — Supplementary Information [file 41598_2017_3723_MOESM1_ESM.pdf]

TITLE: Investigating the Life Expectancy and Proteolytic Degradation of Engineered Skeletal Muscle Biological Machines

AUTHORS AND AFFILIATIONS:

Caroline Cvetkovic [1,2,*], Meghan C. Ferrall-Fairbanks [3,*], Eunkyung Ko [1,2], Lauren Grant [1,2], Hyunjoon Kong [4,5], Manu O. Platt [3], and Rashid Bashir [1,2,5]

[1] Department of Bioengineering, University of Illinois at Urbana-Champaign, Urbana, Illinois 61801

[2] Micro and Nanotechnology Laboratory, University of Illinois at Urbana-Champaign, Urbana, Illinois 61801

[3] Wallace H. Coulter Department of Biomedical Engineering, Georgia Institute of Technology and Emory University, Atlanta, Georgia 30332

[4] Department of Chemical and Biomolecular Engineering, University of Illinois at Urbana-Champaign, Urbana, Illinois 61801

[5] Carle Illinois College of Medicine, University of Illinois at Urbana-Champaign, Urbana, Illinois 61801

For RB, Email: rbashir@illinois.edu. Phone: (217) 333-1867; Fax: (217) 244-6375; Mailing Address: 1270 DCL, 1304 W. Springfield Ave., Urbana, Illinois 61801

For MOP, Email: manu.platt@bme.gatech.edu. Phone: (404) 385-8531; Fax: (404) 385-8109; Mailing Address: 950 Atlantic Drive, Suite 3015, Atlanta, Georgia 30332

[*] *These authors contributed equally to this work.*

SUPPLEMENTARY FIGURES

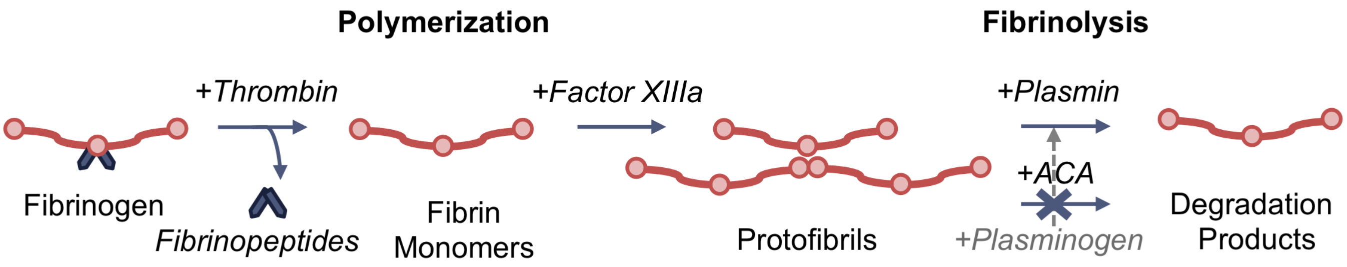

Supplementary Figure S1. Polymerization and Degradation of Fibrin. Fibrinogen is cleaved by thrombin to produce fibrin monomers that can be polymerized into protofibrils. The addition of the protease plasmin can lead to hydrolysis of the fibrin branching network, yielding degradation products. However, the anti-fibrinolytic inhibitor aminocaproic acid (ACA) can be added to prevent plasmin from becoming its active form. ACA binds to the lysine binding sites present on both plasmin and its activating agent plasminogen, which can be produced by C2C12s. ACA prevents plasmin from being activated and binding to (and subsequently degrading) fibrin by competitive and stoichiometric inhibition. For more detail, see references[1–5].

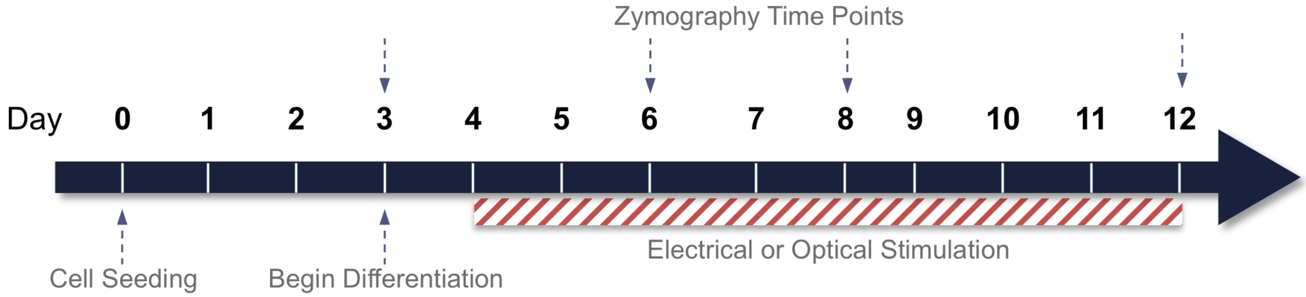

Supplementary Figure S2. Timeline of Bio-Bot Development and Analysis. Skeletal muscle bio-bots were fabricated and seeded on day 0. On day 3, DM was added to induce differentiation of skeletal muscle. Electrical or optical stimulation was applied on days 4-12, as noted. Samples were snap frozen for gelatin zymography on days 3, 6, 8, and 12.

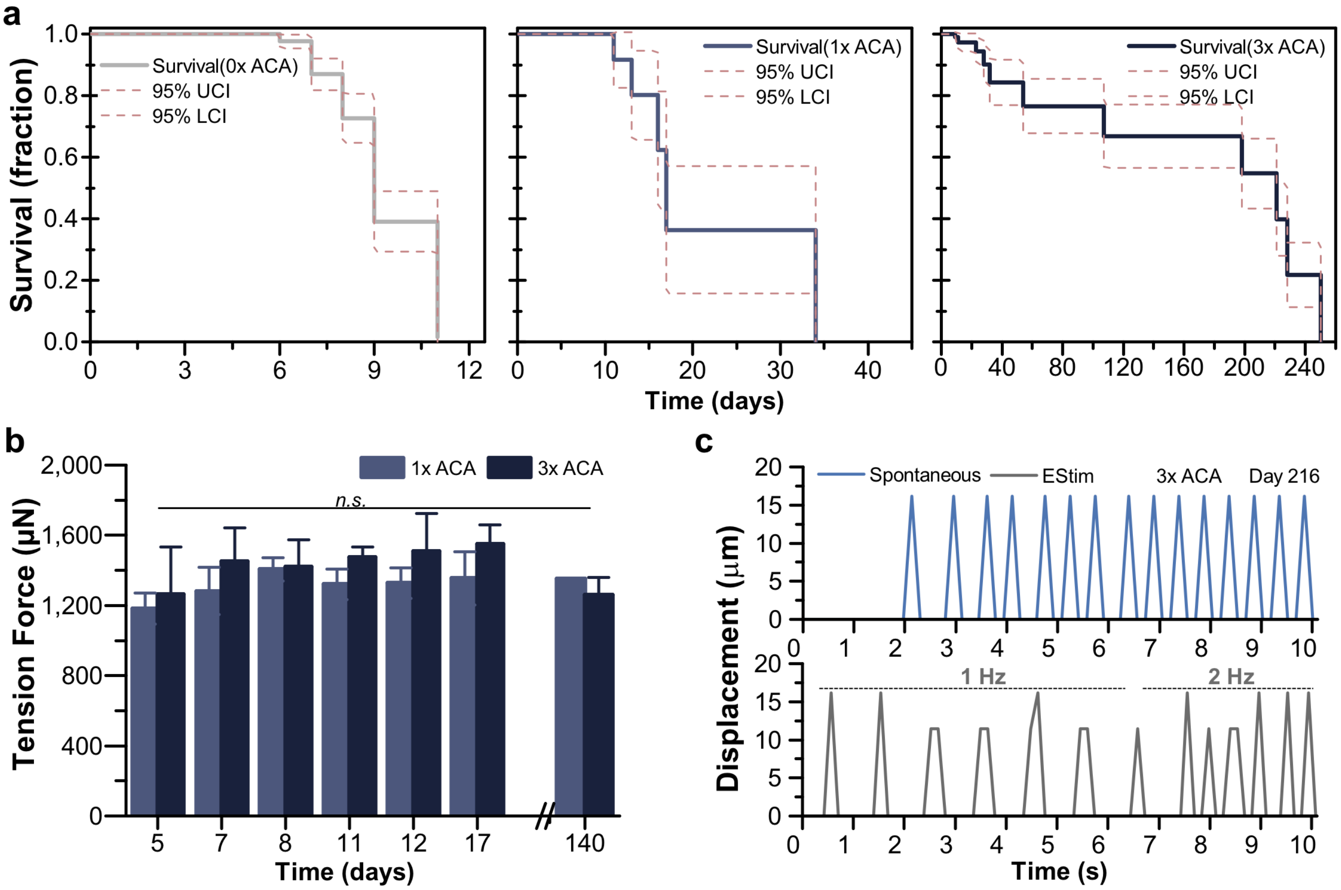

Supplementary Figure S3. Survival Analysis of Bio-Bots with Varying ACA. (a) Kaplan-Meier survival curves are shown for each treatment group (0x, 1x, 3x ACA) as a function of time. 95% confidence intervals (CI) are also shown (*n* = 6-13 muscle strips per condition, excluding outliers). **(b)** Long term, muscle strips cultured with 1x (*n* = 1) or 3x (*n* = 2) ACA maintained passive tension forces that were statistically unchanged on day 140 compared to early values. Plot represents mean ± SEM. **(c)** Muscle strips treated with 3x ACA continued to contract spontaneously (*top*) and in response to electrical stimulation (at 1 Hz and 2 Hz, *bottom*). Plots represent displacement of one pillar of the bio-bot skeleton (see **Supplementary Videos**).

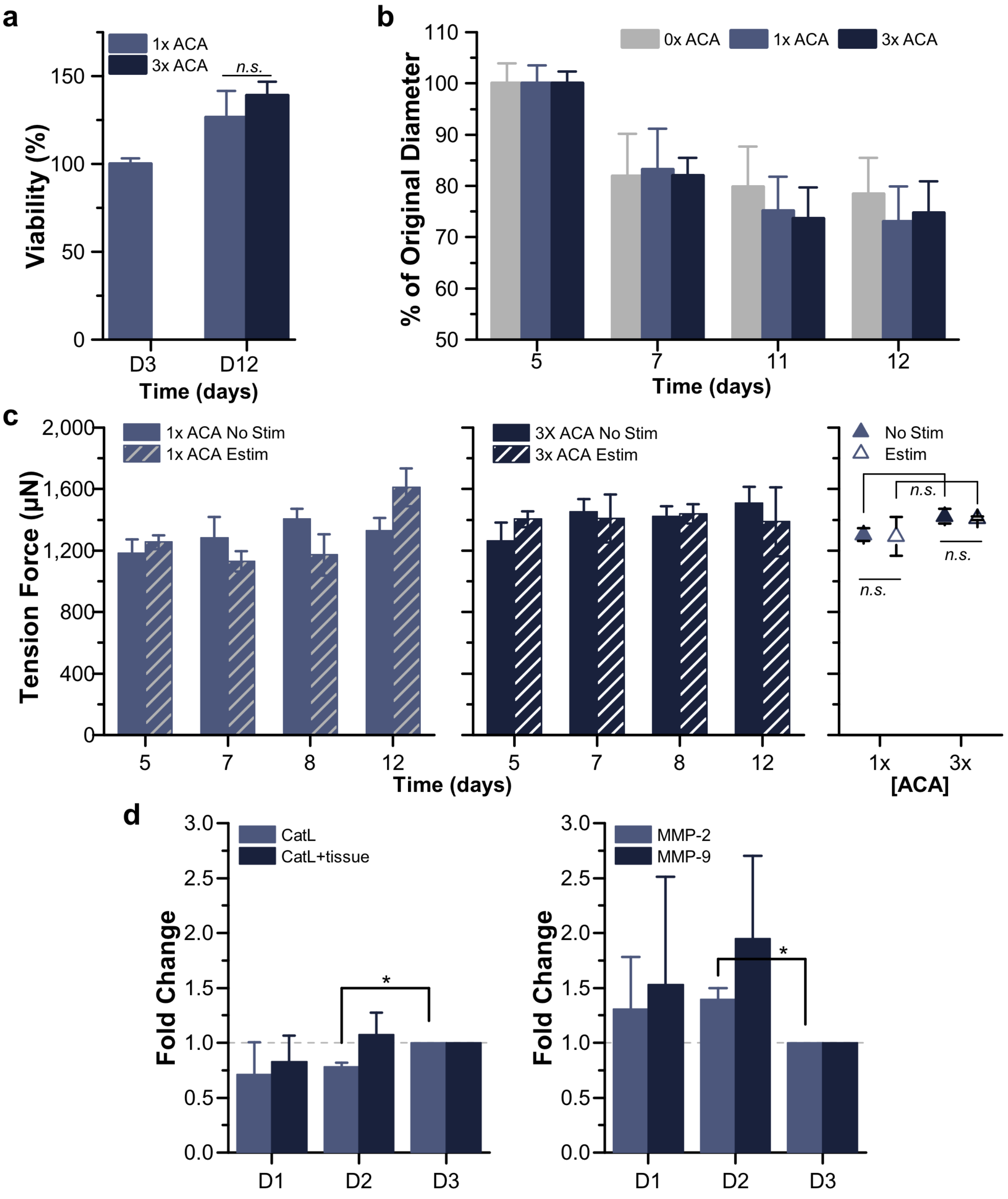

Supplementary Figure S4. Tissue Structure and Mechanical Integrity with ACA. (a) Increasing the concentration of ACA from 1 to 3 mg ml^{-1} did not adversely affect cell viability within the muscle strips, as quantified using a MTS colorimetric assay (*n* = 6-7 muscle strips per condition). The number of living cells was assumed to be directly proportional to the absorbance. **(b)** Average muscle strip tissue diameter as a function of time and ACA treatment, expressed as a percentage of original tissue diameter (*n* = 2-9

muscle strips per condition). **(c)** Muscle strip passive tension for 1x and 3x ACA, with and without electrical stimulation, over time. There was no significant difference in measured tension force across ACA treatment groups or stimulation regimens (n = 5-10 muscle strips per time point). **(d)** Gel zymography identified the amount of active CatL, CatL+tissue, MMP-2, and MMP-9 on days 1-3 (before the start of differentiation). All plots represent mean ± SEM. * indicates significance ($p < 0.05$) between conditions at the same time point.

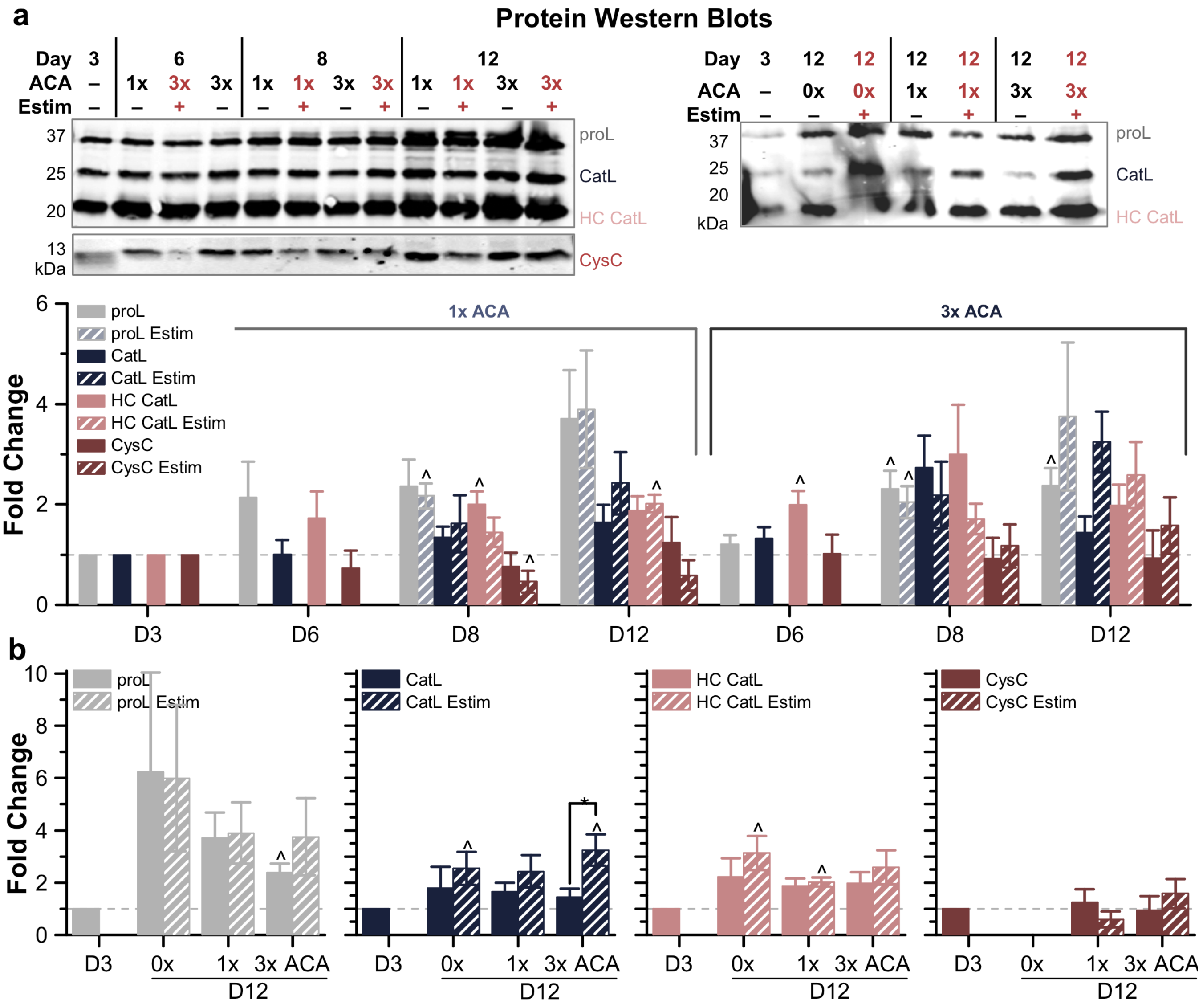

Supplementary Figure S5. Western Blotting of CatL in Muscle Strips with ACA. (a) Western blotting confirmed the presence of cathepsins and related proteins in muscle strips cultured with 1x and 3x ACA over time. **(b)** Western blotting of CatL, with and without electrical stimulation, for day 12 conditions compared to the initial time point of day 3 (n = 4-7 muscle strips per condition). All plots represent mean ± SEM. * indicates significance ($p < 0.05$) between conditions at the same time point; ^ indicates significance compared to initial time point.

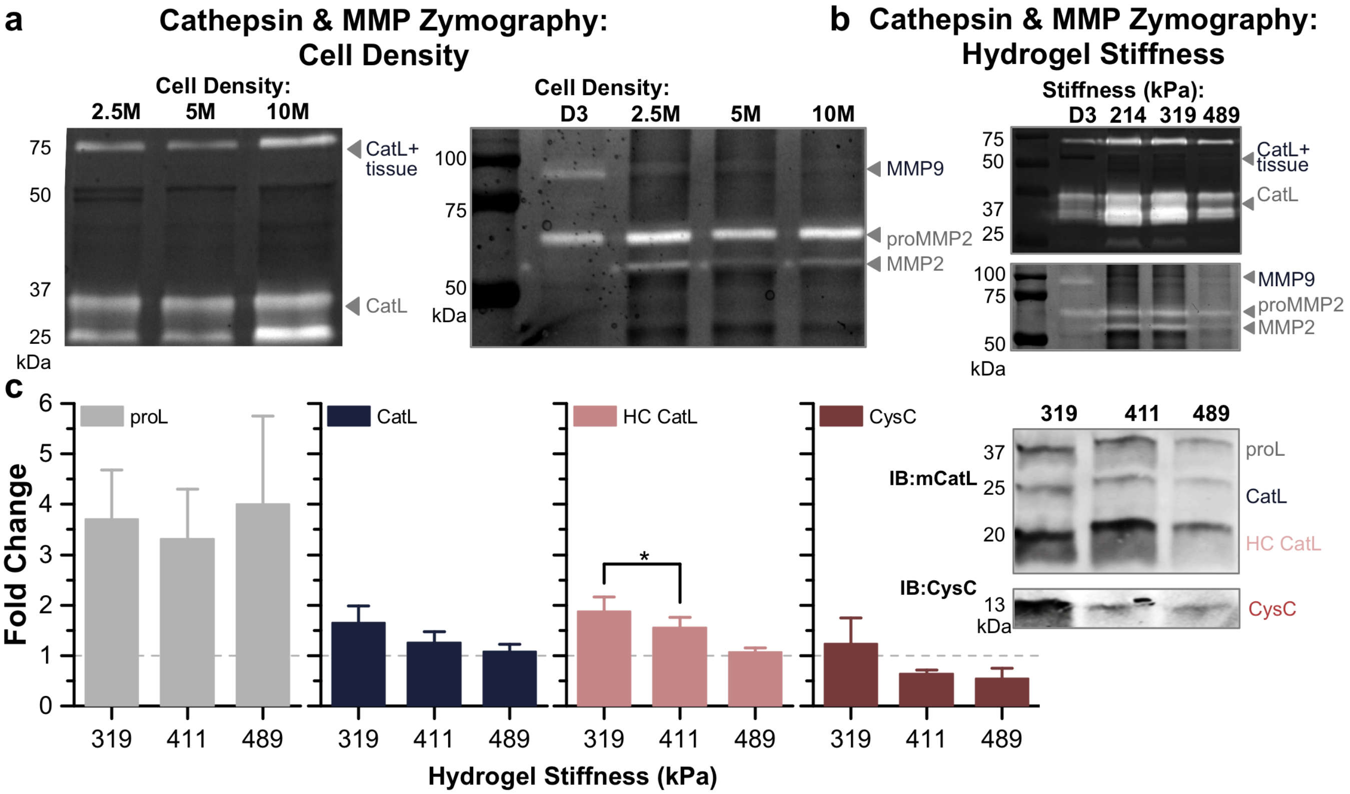

Supplementary Figure S6. Gelatin Zymography of Cathepsins and MMPs in Muscle Strips with Varying Cell Density and Hydrogel Skeleton Stiffness. (a) For day 12 muscle strips cultured with 1x ACA and fabricated with varying cell densities (2.5, 5, or 10 x 10^6 cells ml^{-1}), cathepsin zymography identified the amount of active CatL and CatL+tissue, and MMP zymography identified the amount of active MMP-2 and MMP-9 (n = 7-10 muscle strips per condition) **(b)** For day 12 muscle strips cultured with 1x ACA and with hydrogel skeleton stiffness values of 319, 411, or 489 kPa, cathepsin zymography identified the amount of active CatL and CatL+tissue, and MMP zymography identified the amount of active MMP-2 and MMP-9 (n = 9-17 muscle strips per condition). **(c)** Western blotting confirmed the presence of cathepsins in muscle strips with varying hydrogel stiffness, for day 12 conditions compared to the initial time point of day 3 (n = 3-4 muscle strips per condition). All plots represent mean ± SEM. * indicates significance ($p < 0.05$) between conditions at the same time point.

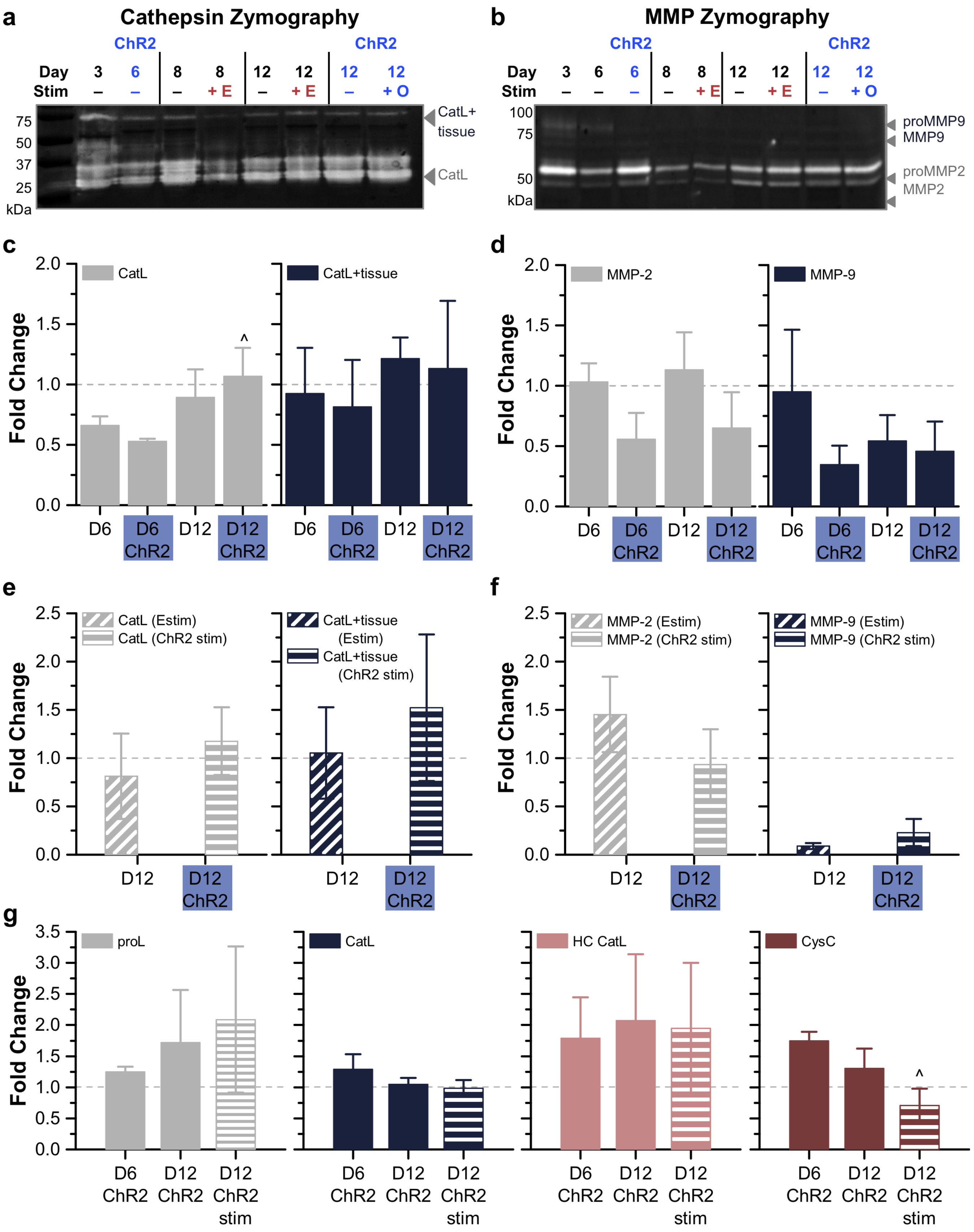
a
Cathepsin Zymography
ChR2
ChR2
Day 3 6 8 8 12 12 12 12
Stim – – – + E – + E – + O
75
50
37
25
kDa
CatL+ tissue
CatL
b
MMP Zymography
ChR2
ChR2
Day 3 6 6 8 8 12 12 12 12
Stim – – – – + E – + E – + O
100
75
50
kDa
proMMP9
MMP9
proMMP2
MMP2
c
CatL
CatL+tissue
Fold Change
D6
D6 ChR2
D12
D12 ChR2
d
MMP-2
MMP-9
Fold Change
e
CatL (Estim)
CatL (ChR2 stim)
CatL+tissue (Estim)
CatL+tissue (ChR2 stim)
Fold Change
D12
D12 ChR2
f
MMP-2 (Estim)
MMP-2 (ChR2 stim)
MMP-9 (Estim)
MMP-9 (ChR2 stim)
Fold Change
g
proL
CatL
HC CatL
CysC
Fold Change
D6 ChR2
D12 ChR2
D12 ChR2 stim

Supplementary Figure S7. ChR2-C2C12 Optogenetic Bio-Bots. (a) Cathepsin zymography identified the amount of active CatL and CatL+tissue and **(b)** MMP zymography identified the amount of active MMP-2, and MMP-9, with and without electrical or optical stimulation. **(c)** Amount of active CatL, CatL+tissue, **(d)** MMP-2, and MMP-9 on days 6 and 12, for both non-optogenetic bio-bots as well as ChR2-C2C12 bio-bots without optogenetic stimulation. **(e)** Amount of active CatL, CatL+tissue, **(f)** MMP-2, and MMP-9 on day 12, for both non-optogenetic bio-bots subjected to electrical stimulation and ChR2-C2C12 bio-bots subjected to optical stimulation. 3 **(g)** Western blotting confirmed the presence of cathepsins in ChR2-C2C12 optogenetic muscle strips on days 6 and 12, with and without optical stimulation (n = 3 muscle strips per condition). All plots represent mean ± SEM. ^ indicates significance ($p < 0.05$) compared to initial time point (day 6 for panels c, d, and g).

SUPPLEMENTARY VIDEOS

Supplementary Video 1. Spontaneous Contraction after 7 Months. Muscle strips treated with 3x ACA contracted spontaneously after 7 months of culture.

Supplementary Video 2. Electrical Stimulation after 7 Months. Muscle strips treated with 3x ACA responded to electrical pulse stimulation (1 Hz and 2 Hz) after 7 months of culture.

REFERENCES

1. Kupcsik, L., Alini, M. & Stoddart, M. J. Epsilon-aminocaproic acid is a useful fibrin degradation inhibitor for cartilage tissue engineering. *Tissue Eng. Part A* **15,** 2309–2313 (2009).
2. Prentice, C. R. M. Basis of antifibrinolytic therapy. *J. Clin. Pathol.* **14,** 35–40 (1980).
3. Janmey, P. A., Winer, J. P. & Weisel, J. W. Fibrin gels and their clinical and bioengineering applications. *J. R. Soc. Interface* **6,** 1–10 (2009).
4. Weisel, J. W. The mechanical properties of fibrin for basic scientists and clinicians. *Biophys. Chem.* **112,** 267–76 (2004).
5. Weisel, J. W. in *Advances in Protein Chemistry* (eds. Parry, D. A. D. & Squire, J. M.) **70,** 247–299 (Academic Press, 2005).
